# Epigallocatechin gallate regulates the myeloid-specific transcription factor PU.1 in macrophages

**DOI:** 10.1371/journal.pone.0301904

**Published:** 2024-04-25

**Authors:** Manjula Karpurapu, Kavita Kumari Kakarala, Sangwoon Chung, Yunjuan Nie, Amritendu Koley, Patrick Dougherty, John W. Christman

**Affiliations:** 1 Division of Pulmonary, Davis Heart and Lung Research Institute, Critical Care and Sleep Medicine, Ohio State University Wexner Medical Center, Columbus, OH, United States of America; 2 Mahavir Hospital and Research Center, Lakdikapul, Hyderabad, Telangana, India; 3 Department of Basic Medicine, Wuxi School of Medicine, Jiangnan University, Wuxi, Jiangsu, 214122, P.R. China; 4 Department of Chemistry and Biochemistry, Ohio State University, Columbus, OH, United States of America; Jeju National University, REPUBLIC OF KOREA

## Abstract

Our previous research demonstrated that PU.1 regulates expression of the genes involved in inflammation in macrophages. Selective knockdown of PU.1 in macrophages ameliorated LPS-induced acute lung injury (ALI) in bone marrow chimera mice. Inhibitors that block the transcriptional activity of PU.1 in macrophages have the potential to mitigate the pathophysiology of LPS-induced ALI. However, complete inactivation of PU.1 gene disrupts normal myelopoiesis. Although the green tea polyphenol Epigallocatechin gallate (EGCG) has been shown to regulate inflammatory genes in various cell types, it is not known if EGCG alters the transcriptional activity of PU.1 protein. Using Schrodinger Glide docking, we have identified that EGCG binds with PU.1 protein, altering its DNA-binding and self-dimerization activity. *In silico* analysis shows that EGCG forms Hydrogen bonds with Glutamic Acid 209, Leucine 250 in DNA binding and Lysine 196, Tryptophan 193, and Leucine 182 in the self-dimerization domain of the PU.1 protein. Experimental validation using mouse bone marrow-derived macrophages (BMDM) confirmed that EGCG inhibits both DNA binding by PU.1 and self-dimerization. Importantly, EGCG had no impact on expression of the total PU.1 protein levels but significantly reduced expression of various inflammatory genes and generation of ROS. In summary, we report that EGCG acts as an inhibitor of the PU.1 transcription factor in macrophages.

## Introduction

The ETS family transcription factor PU.1 encoded by the Sfpi1 gene, regulates the development of myeloid lineage cells from hematopoietic progenitors. PU.1 is required for the selective development of the lymphoid or myeloid lineages and is downregulated during erythropoiesis [1–4]. Expression levels of PU.1 play an essential role in hematopoiesis and hematopoietic cell lineage specification. Decreased expression levels of PU.1 favor normal development of megakaryocyte-erythroid progenitors, B cell, and T cell progenitors, whereas increased levels of PU.1 favor granulocyte/macrophage progenitors [2,4,5]. Decreasing expression levels of PU.1 protein in the myeloid lineages leads to impaired differentiation, abnormal proliferation, and leukemia [6–8]. Furthermore, macrophages from the lungs of patients with pulmonary alveolar proteinosis are relatively deficient in PU.1 [9]. PU.1 is involved in the transcriptional regulation of the GM-CSF receptor, required for the terminal differentiation of macrophages [10–12] and its deficiency impairs innate and acquired host immunity by partly reducing the cellular expression levels of TLR2 and TLR4, which are essential in pathogen recognition [13–16]. More importantly, high throughput studies have demonstrated a genome-wide association of PU.1 with inflammatory stimuli-induced gene expression patterns [17–19]. Expression of CD11b and other endotoxin signaling genes, including TREM1, TLR2, and TLR4 are transcriptionally regulated by PU.1 [14,16]. These genes regulate the initiation and intensity of the endotoxin response in macrophages [20,21]. In humans subjected to experimental endotoxemia, a 2- to 3-fold increase in CD 11b, TLR4, and TLR2 expression is observed in circulating blood monocytes, implicating the involvement of PU.1 in inflammatory signaling [22].

Our prior work using bone marrow chimeras of NF-κB luciferase reporter mice conditionally expressing Tamoxifen inducible PU.1 protein in macrophages demonstrated that myeloid-specific PU.1 protein is pivotal in regulating NF-κB dependent acute lung inflammation and macrophage TLR4 gene expression during endotoxemia [23]. We also demonstrated that PU.1 regulates monocyte-to-macrophage differentiation [24]. Although the critical role of PU.1 protein in myeloid lineage specification, inflammatory signaling, and leukemias is well studied, no pharmacological inhibitors have been developed. Heterocyclic di-amidines and di-cations were designed that act as small molecule inhibitors of PU.1 DNA binding [25,26]. Based on the mechanism of action, the heterocyclic di- amides and di-cations bind to genome-wide minor groove DNA, which in turn inhibits the binding of PU.1 to DNA [27,28]. In the current study, we used a molecular docking approach and demonstrated that EGCG acts like a small molecule structural inhibitor that binds to PU.1 protein.

EGCG is the most abundant polyphenol found in green tea extracts. Prior research indicates that EGCG regulates various cellular targets, particularly in the context of cancer, neurodegenerative, and metabolic disorders [29–31]. From a mechanistic standpoint, EGCG influences various cellular processes in distinct ways. EGCG modulates signaling pathways such as PI3K/Akt, MAPK, NF-κB, and Wnt/β-catenin, which are critical for cancer cell survival, proliferation, and metastasis [32]. In addition, EGCG increases the activity of antioxidant enzymes which protect cells against oxidative damage [33]. Furthermore, EGCG reduces blood glucose amounts, improves insulin resistance and islet β-cell function in type 2 diabetes [34]. EGCG also inhibits NF-κB and MAPK signaling pathways in macrophages, protects endothelial cells by decreasing the expression levels of adhesion molecules, enhances tight junction protein expression maintaining the epithelial barrier function in acute lung injury [35,36]. Recent studies have also revealed EGCG’s potent antiviral activity, inhibiting viral entry and replication of SARS-CoV-2 in host cells [37]. Our study has expanded on the current understanding of the cellular effects of EGCG, identifying a novel mechanism through which EGCG inhibits DNA binding and dimerization activities of PU.1, consequently reducing the expression of key inflammatory genes and ROS generation in macrophages.

## Materials and methods

### Molecular docking

The structure of EGCG ligand was downloaded from PubChem database (http://www.ncbi.nlm.nih.gov/pccompound). Ligand preparation tool, Ligprep (LigPrep, version 2.5, Schrödinger, LLC, New York, NY) was used to prepare ligand that included conversion from 2D to 3D, structure corrections, generation of ionized state, tautomers and optimization of the ligand structures.

#### Protein preparation and docking

The crystal structure of DNA bound mouse PU.1 (PDBID 1PUE) was downloaded from PDB database (www.rcsb.org). For docking, crystal structure of 1PUE was prepared using protein preparation wizard of Schrödinger (Schrödinger Release 2013–2: Schrödinger Suite 2013 Protein Preparation Wizard). It included addition of missing hydrogen atoms and correcting bond order assignments, charge states and orientation of various groups, deletion of water molecules and addition of missing sidechain atoms, followed by minimization using OPLS_2005 Force field. The Receptor grid for 1PUE was placed at residues (R232, I172, R173, L174, W215, 219K, 221N, 222M, 229K, 223A. 236N, 237Y, 240T, 242E) identified using SiteMap tool [38]. After the grid generation, the prepared ligands were docked to the protein (1PUE) using GLIDE docking protocol. The ligand was docked using Glide XP mode (GlideXP, Schrödinger, LLC, New York, NY, 2015) at default settings. The docked conformers were evaluated using Glide (G) Score calculated as follows: G Score = a*vdW+b*Coul + Lipo + Hbond + Metal + BuryP + RotB + Site. vdW denotes van der Waals energy, Coul denotes Coulomb energy, Lipo denotes lipophilic contact, HBond indicates hydrogen-bonding, Metal indicates metal-binding, BuryP indicates penalty for buried polar groups, RotB indicates penalty for freezing rotatable bonds, Site denotes polar interactions in the active site and the a = 0.065 and b = 0.130 are coefficients of vdW and Coul in this equation. The binding affinity between the 1PU1 and EGCG was determined as negative values of GLIDE scores (kJ/mol), such that the higher the negative value of the pose the stronger the protein-ligand (PU1: EGCG) interaction.

#### Structural interaction fingerprint (SIFt) analysis

SIFt was used to analyse the common binding mode of the docking poses to identify residues that display polar or non-polar contacts with the docking poses as described previously [39,40]. SIFt is a binary fingerprint representation of the intermolecular interactions of the three-dimensional protein–inhibitor complex. The output of molecular docking studies of PU1 and EGCG were analyzed using this tool of Schrodinger. During the generation of SIFt fingerprints, data related to the proximity of the ligand to PU.1 protein residues and the types of residues participating in the interactions are collected and stored [39]. This information captures the spatial arrangement and characteristics of the ligand-protein interactions, including factors such as hydrogen bonds, hydrophobic contacts, electrostatic interactions, and van der Waals interactions. Furthermore, this tool was used to identify key binding site residues important for specific interactions (hydrophobic, aromatic, charge, polar, side chain, and backbone) (Schrödinger Release 2018: Maestro, Schrödinger, LLC, New York, NY, 2018). Using the same approach, binding residues between homology modeled human PU.1 protein and EGCG were deduced.

#### Bone marrow derived macrophage (BMDM) isolation from C57black6 mice

All animals used for cell isolation were housed at the OSU and used as per Institutional Animal Care and Use Committee (IACUC) approved protocols (2013A00000105-R3) based on the American Veterinary Medical Association Panel guidelines for euthanasia, methods of anesthesia and efforts to alleviate pain. C57black 6 mice were euthanized as per AVMA guidelines with an overdose of anesthetic (300 mg/kg ketamine and 30 mg/kg xylazine), and a secondary method by cervical dislocation. Femurs were separated from euthanized animals, total bone marrow cells were isolated and cultured in DMEM (Thermofisher, Waltham, MA) supplemented with 10% FBS, 50 IU/mL Penicillin and 50 μg/mL Streptomycin. Recombinant mouse M-CSF (20 ng/mL) was added on Day 1, 3 and 5, and BMDM were differentiated up to 7 days as described previously [23,24]. THP-1 cells were purchased from ATCC (#TIB-202), and cultured in RPMI1640, supplemented with 10% FBS, 50 IU/mL Penicillin and 50 μg/mL Streptomycin, β-mercaptoethanol and differentiated to macrophages by adding 200 ng/mL PMA and grown for 48 h.

#### Labeling EGCG with Tetramethyl rhodamine (TRITC)

EGCG is conjugated with TRITC using a previously published protocol [41]. 1% solution of EGCG in 0.2 M K2HPO4 (pH 9.2) was incubated for 72 h at 37° C in the presence of 60 mg TRITC. Following the incubation period, the resulting conjugate was extracted using 100 mL of ethyl acetate, and the solvent was evaporated at 50° C. The resultant material was then dissolved in an excess of double-distilled water, subsequently recovered through the process of freeze-drying and confirmed by HPLC.

#### Fluorescence polarization assay

Purified PU.1 protein was purchased from Origene (Rockville, MD). Binding affinity of EGCG-Rhodamine (50 nM) and PU.1 (0–1000 M) was determined using fluorescence polarization assay as described previously [42]. In brief, PU.1 protein and EGCG-TRITC were incubated in 20 mM HEPES (pH 7.4), 150 mM NaCl, 2 mM Mg(OAc)_2_, and 0.1% bovine serum albumin for 2 h at room temperature. The fluorescence was measured at 525 nM on a Molecular Devices Spectramax M5 Spectrofluorimeter. Dissociation constant was calculated by plotting the FA values as a function of the PU.1 concentration.

#### Cellular uptake of EGCG-TRITC

1 μM EGCG-TRITC was added to mouse BMDM plated on 8 well glass slides and grown for further 1–24 hours. Cells were fixed with 1% paraformaldehyde, mounted and imaged using Olympus FV3000 confocal microscope at Campus microscopy and Imaging facility, Ohio State University.

#### Lactate Dehydrogenase assay

BMDM (2x10^3^ cells/well) were grown on Perkin Elmer 96 well plates (Waltham, MA) in presence of 0–25 μM EGCG for 24 hours. Cellular toxicity was measured by release of LDH using Promega LDH-Glo™ Cytotoxicity Assay kit (Madison, WI) as per the manufacturer’s instructions.

#### ROS assay

Cellular ROS levels were assayed using Cell Biolabs OxiSelect™ Intracellular ROS Assay Kit (San Diego, CA) to measure conversion of 2’, 7’-Dichlorodihydrofluorescin diacetate (DCFH-DA) to DCF, as per the manufacturer’s instructions.

#### PU.1 DNA binding assays

DNA binding was assayed by electrophoretic mobility shift assay (EMSA) and chromatin immunoprecipitation (ChIP) as described previously [24,43].

#### EMSA

5’Biotin labeled TLR4 promoter oligo was purchased from IDT DNA (Coralville, IA). Cellular nuclear protein fractions were extracted by Pierce Nuclear and Cytoplasmic Extraction Reagents kit (Waltham, MA). Nuclear extracts were incubated with biotin-labeled TLR4 promoter oligo, electrophoresed on 7.5% native polyacrylamide gel in 1XTAE buffer, and transferred on to Biodyne A membrane. The DNA-protein complexes on the membrane were detected by Pierce biotin-streptavidin HRP chemiluminescence detection kit (Waltham, MA).

#### ChIP

PU.1 bound chromatin from BMDM cells was processed using Qiagen EpiTect ChIP OneDay Kit (Germantown, MD) and PCR amplified using primers (forward 5’ ACT CTC ACT TCC TCT TTG AAT ATA3’; reverse 5’ TAT ATT CAA AGA GAA GTG AGA GT3’) that encompass PU.1 binding site at -90 bp region from transcription start site of TLR4. Differences in PU.1 binding to TLR4 promoter (amount of DNA immuno-precipitated by PU.1 antibody) was expressed as % of input DNA (total DNA) at the start of the experiment.

#### Western blotting

BMDM were pre-treated with ethanol or 5 μM of EGCG for 1 h and stimulated with LPS or PBS for another 16 h. BMDM were lysed with 1X RIPA buffer, electrophoresed on 4–20% polyacrylamide gel and transferred on to PVDF membrane. The membranes were incubated with primary antibodies (Cell signaling technologies, Danvers, MA) against PU.1, COX2, iNOS, and β-actin and host specific secondary antibodies, as described previously [43]. To determine the monomeric or dimeric states of PU.1, 30 μg of total cell lysate from BMDM was incubated with 25 μM EGCG (without heating) and β-mercapto ethanol (heated at 95°C for 2 minutes) separately and electrophoresed on 4–20% native PAGE. Native-PAGE separated proteins were transferred on to PVDF membrane and immunoblotted with anti-PU.1. Levels of immunoreactive proteins were visualized using Pierce chemiluminescence kits (Waltham, MA)

### Quantitative real time PCR

Total RNA from the BMDM were isolated using Direct-zol RNA Kit that includes a genomic DNA removal step as per the manufacture’s instruction (Zymo Research, Irvine, CA). cDNA was synthesized from total RNA, using RevertAid First Strand cDNA Synthesis Kit (Thermofisher Scientific, Waltham, MA) and gene expression was measured by quantitative PCR on Roche LightCycler 480. Target gene expression levels were quantified using 2(^−ΔΔ^Ct) method normalized to GAPDH expression. Different target gene primers used for qPCR are:

Mouse GAPDH forward 5′- tgg aac aag gag gag cag aga gca-3′, reverse 5′-tac tcg cgg ctt tac ggg-3′; CCR2 forward 5’-tgc aaa aac aaa tga aag gga agg-3’, reverse 5’-gga gta gag tgg agg cag ga-3’; COX2 forward 5’-gcc cag cac ttc acc cat cag t-3’, reverse 5’-aag tcc act, cca tgg ccc agt cc-3’; iNOS forward 5’-ccg ccc tgg tgc agg gaa tc-3’, reverse 5’-acc cag tag cg ccg ctc tca-3’; IL6 forward 5’-agt, ccg, gag, agg, aga, ctt, ca-3’, reverse 5’-ttg cca ttg cac aac tct tt-3’; KC forward 5′-tcg cca atg agc tgc gct gtc-3′, reverse 5′-gct tca ggg tca agg caa gcc-3′; TNF-α forward 5′-ttc tca ttc ctg ctt gtg g-3′, reverse, 5′-act tgg tgg ttt gct acg-3′.

Human GAPDH forward 5’-gaa ggt gaa ggt cgg agtc-3’, reverse 5’-gaa atg gtg atg gga ttg-3’; IL6 forward 5’-aaa ttc ggt aca tcc tcg acgg-3’, reverse 5’-gga agg ttc agg ttg ttt tct gc-3’; IL8 forward 5’-act gag agt gat tga gag tgg ac-3’ reverse 5’-aac cct ctg cac cca gtt tc-3’.

### Statistical analyses

All the measurements were expressed as mean±SEM and the statistical differences between single comparisons were performed by Student’s t-test, and the differences between multiple groups by non-parametric ANOVA Kruskall-Wallis test using GraphPad Prism 9. Statistical significance was defined as as */# p< 0.05, **/##p <0.01, ***/###p<0.001, ****/####p<0.001.

## Results

### Identifying EGCG as a structural inhibitor of PU.1 protein

**Fig 1:**
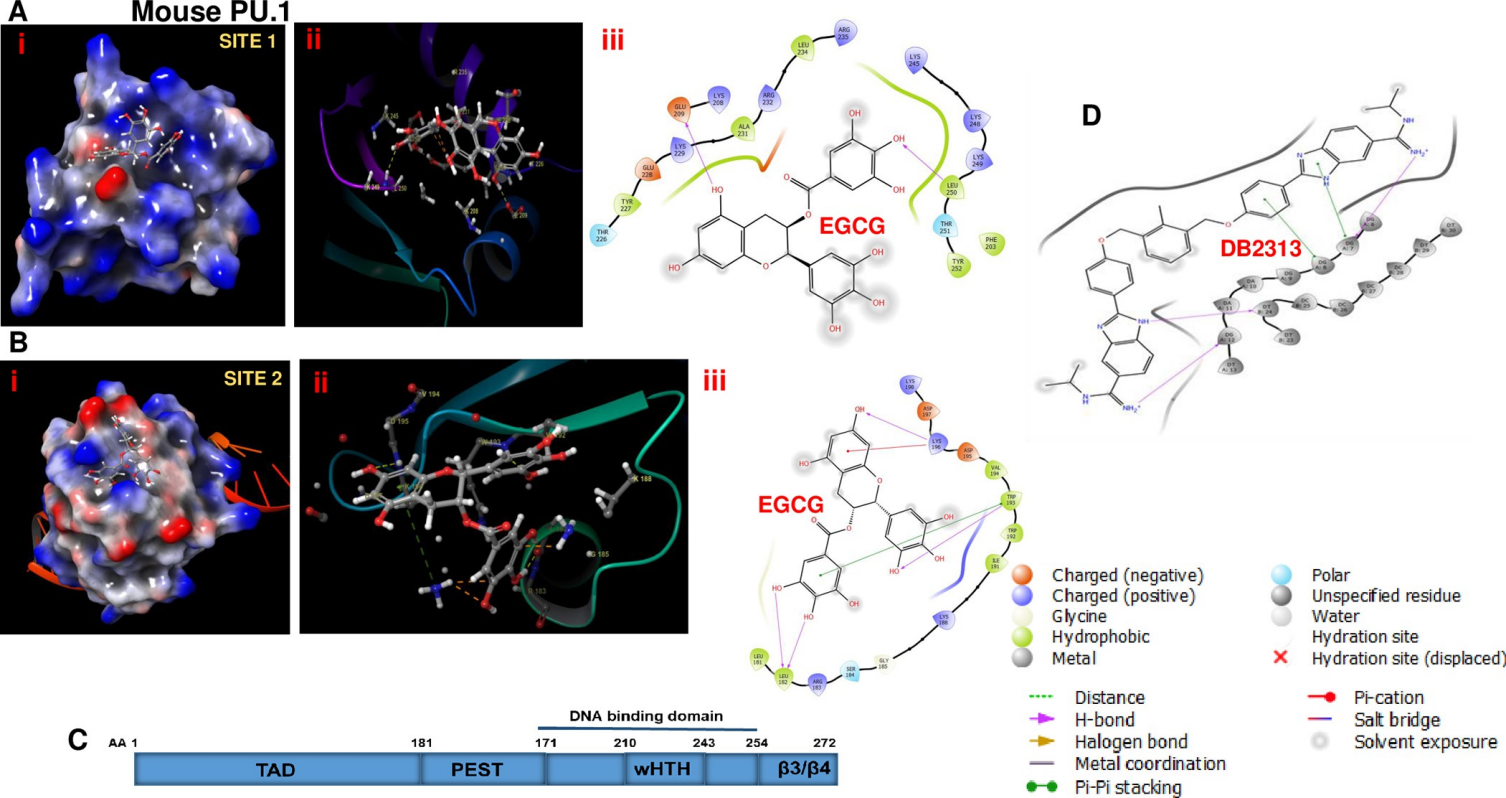
Molecular docking shows specific interaction of EGCG and mouse PU.1 protein. **A)** EGCG was docked on to mouse PU.1 protein 3D model using Schrodinger GLIDE® and protein-ligand structure binding affinity is determined. EGCG establishes hydrogen bonds with Glutamic acid 209, Leucine 250 in the DNA binding domain, and **B)** Leucine 182, tryptophan 193, lysine 196 in the dimerization domain of PU.1 protein. **C)** Structural organization of mouse PU.1 protein **D)** Intercalation of DB2313 with double stranded DNA minor groove.

**Fig 2:**
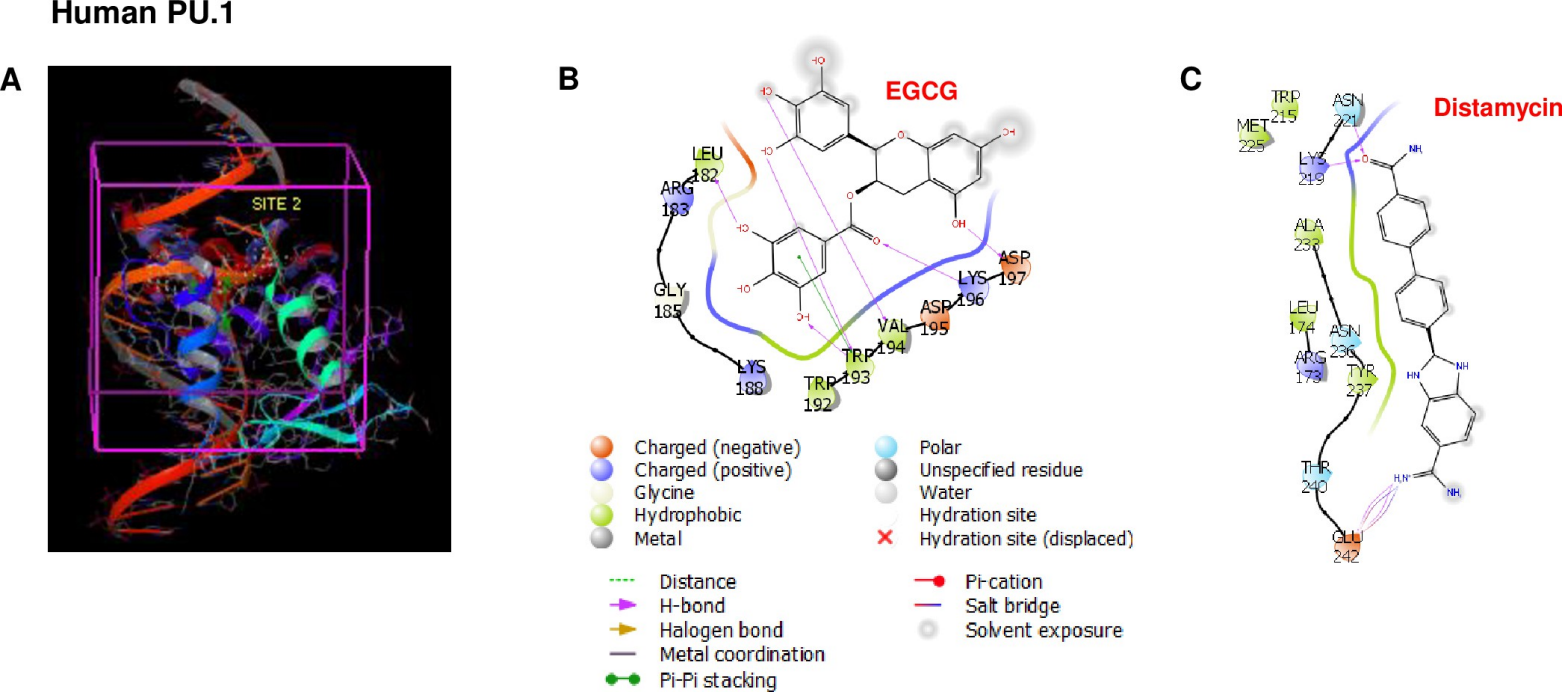
Molecular docking shows specific interaction of EGCG and human PU.1 protein. **A)** EGCG was docked on to homology modeled human PU.1 protein 3D model using Schrodinger GLIDE® and protein-ligand structure binding affinity is determined. **B)** EGCG establishes hydrogen bonds with Leucine 182, tryptophan 193, lysine 196 in the dimerization domain of PU.1 protein. **C)** Interaction of homology modeled human PU.1 protein and Distamycin.

Using Schrodinger Glide® EGCG was docked against mouse PU.1 protein to generate protein ligand complex structures and most favorable binding mode of the PU.1 and EGCG was selected as described previously [40]. Molecular docking analysis indicated that both Glutamic acid 209 and Leucine 250 act as Hydrogen bond acceptors in the DNA binding domain with a Glide score of -9.095935 and glide energy of -26.697188, suggesting a strong affinity between EGCG and mouse PU.1 protein DNA binding domain (Fig 1A). In addition, leucine 182, tryptophan 193, lysine 196 act as Hydrogen bond acceptors in the dimerization domain of PU.1 protein with a Glide score of -8.06 (Fig 1B). Structural organization of PU.1 protein is illustrated in Fig 1C for reference. DB2313 is a commercially available, heterocyclic diamidine that specifically disrupts binding of PU.1 to chromatin through interaction with the DNA bases in minor groove similar to Distamycin [25,28], illustrated in Fig 1D. Human and Mouse PU.1 proteins show high degree of sequence homology, conserved amino acids in the DNA binding and dimerization domains, possibly indicating a similar mode of inhibition of PU.1 in mouse and human cells (S1 Fig). We have further analyzed the interaction of EGCG and human PU.1 protein dimerization domain that showed a docking score of -7.9 and ligand amino acid Hydrogen bonds similar to that of mouse PU.1 protein (Fig 2A and 2B). Glide score of human PU.1 DNA binding domain with EGCG was -9.08. Both Distamycin and DB2313 allosterically interfere with PU.1-chromatin binding through interaction with the DNA minor groove that flanks PU.1-binding sites. Asn 221 and Lys 219 indicated formation of Hydrogen bonds with Distamycin with low affinity (Fig 2C). This indicates that both DB2313 and distamycin inhibit the transcriptional activity of PU.1 by intercalating in the promoter DNA region.

### TRITC-EGCG conjugation to assay EGCG-PU.1 binding

**Fig 3:**
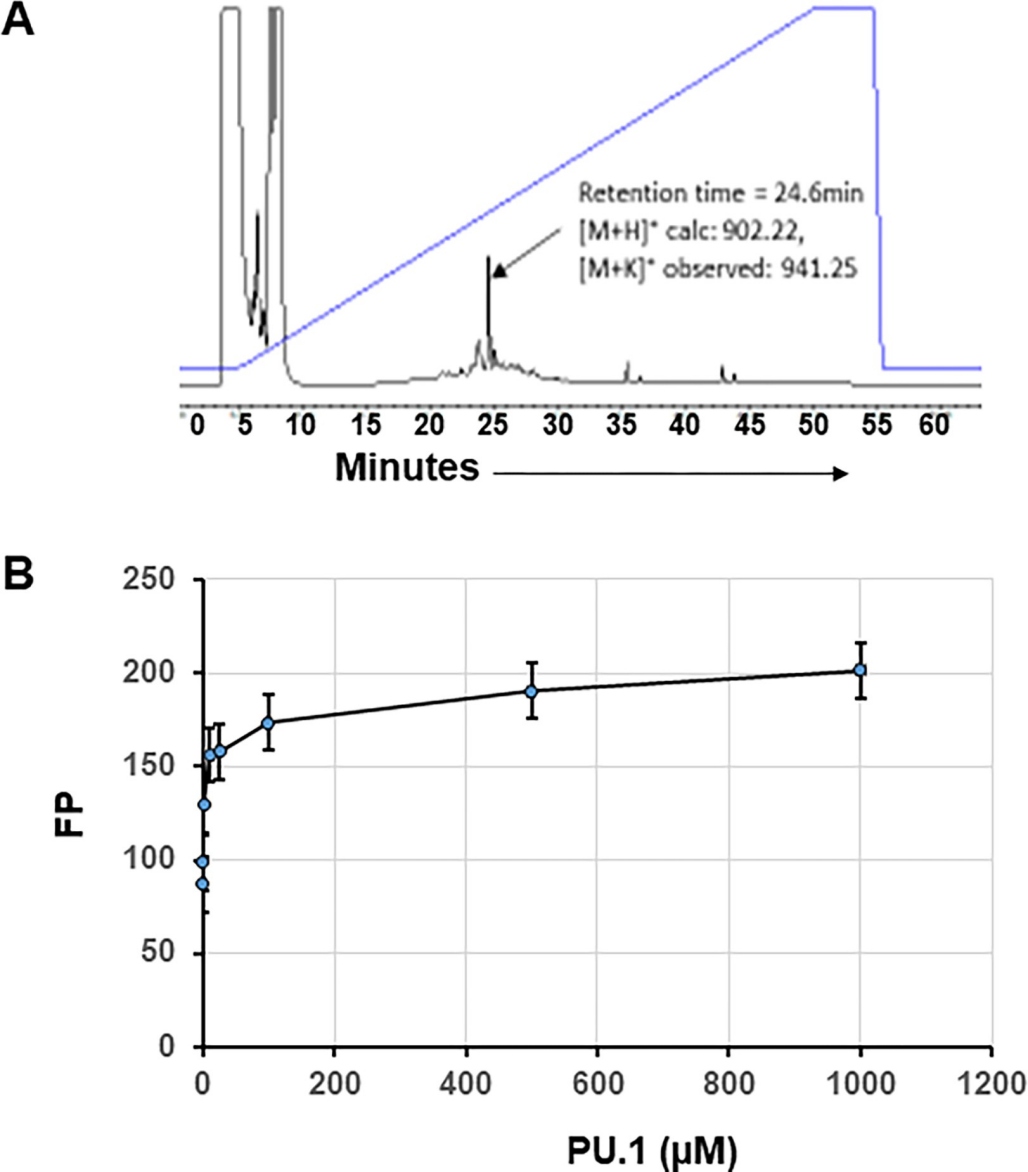
Labeling EGCG with TRITC and determination of EGCG binding affinity. **A)** TRITC was conjugated to EGCG as described in methods. EGCG-TRITC was eluted as a single peak by HPLC and the molecular mass of 940 is equivalent to the cumulative mass of EGCG and TRITC together. **B)** Binding affinity of EGCG-TRITC to PU.1 protein determined by fluorescence anisotropy and calculated dissociation constant of 2.8 μM.

To determine cellular uptake and binding affinity calculations, TRITC conjugated EGCG is prepared using a published protocol [40]. EGCG-TRITC showed an expected molecular mass around 900 Da, which is equivalent to the sum of EGCG and TRITC molecular masses as determined by HPLC (Fig 3A). The binding affinity of PU.1 to EGCG-TRITC was assayed by fluorescence polarization assay as described previously [42]. 0–1000 μM of PU.1 protein was incubated with 50 nM TRITC labeled EGCG and FA was measured, which showed a dose dependent binding. The kD is around 2.8 μM, calculated from the fluorescence polarization assay (Fig 3B).

### Cellular uptake of EGCG-TRITC by BMDM

**Fig 4:**
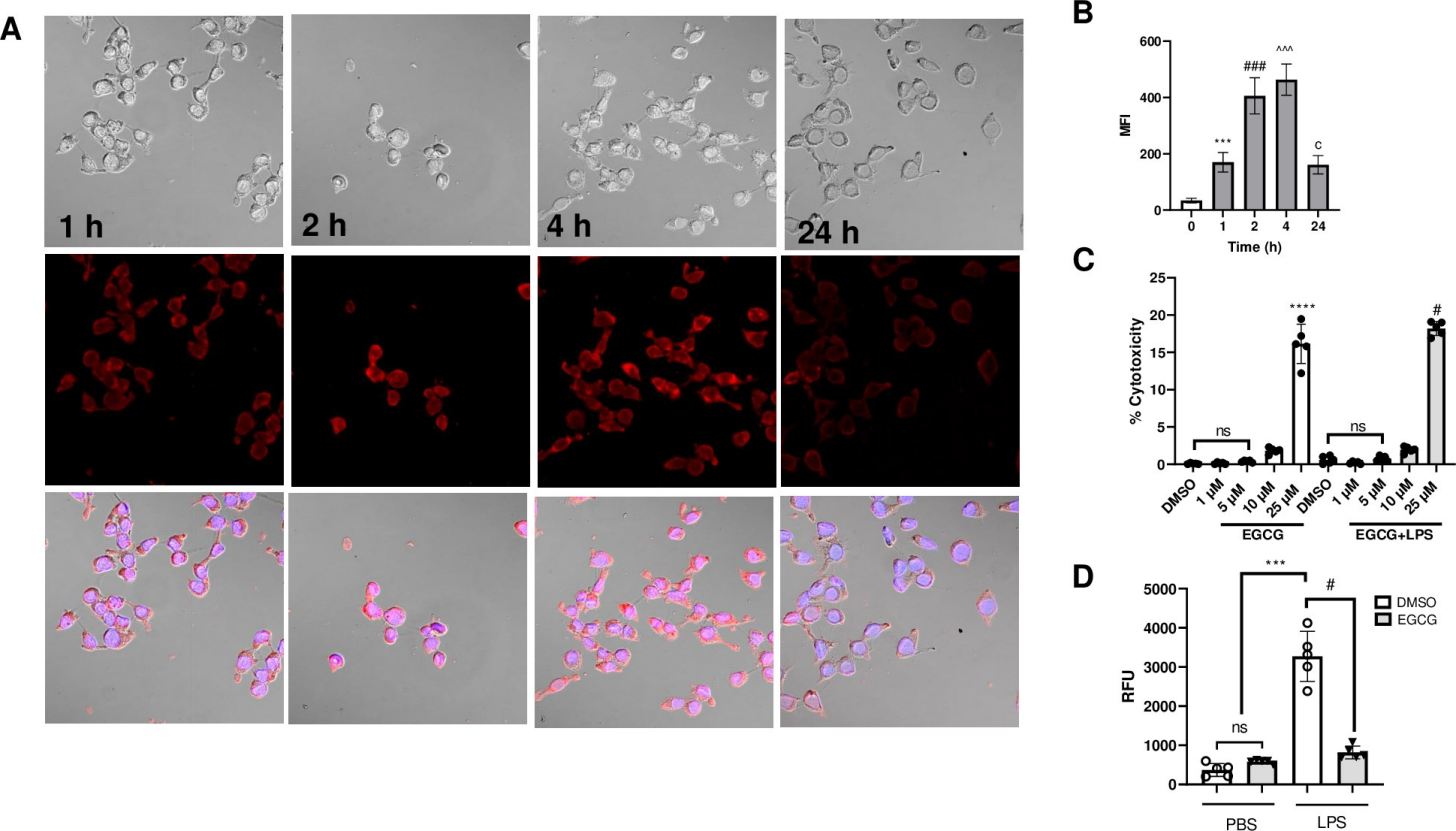
Cellular uptake of EGCG alters ROS generation in BMDM. **A)** Mouse BMDM were pretreated with 5 μM of EGCG-TRITC for the indicated time periods and cellular localization of EGCG-TRITC (PE) is imaged using FV3000 Olympus confocal microscope. **B)** Relative mean fluorescence intensity of PE positive BMDM. **C)** Cellular toxicity is assayed by measuring LDH concentration by pretreating the cells with 1–25 μM EGCG.**D)** Mouse BMDM were pretreated with 5 μM of unlabeled EGCG and LPS-induced ROS generation in BMDM is measured using the DCFH dye as described in methods. Data are shown as mean ± SEM. N = 5 for each group, **B** ***/ ### /^^^ / c p < 0.001 control vs TRITC EGCG **C** ****/ # p < 0.0001 25 μM EGCG vs DMSO or 1, 5, 10 μM EGCG; **D** *** p < 0.001 LPS vs PBS or EGCG alone # p < 0.01 DMSO+LPS vs EGCG+LPS.

Cellular uptake of EGCG-TRITC by mouse BMDM was determined by confocal microscopy using FV3000 Olympus Microscope. As shown in Fig 4A and 4B, 1 μM EGCG is taken up by BMDM within 1 hr of incubation, maximum uptake of EGCG is observed around 4 hours. Most of the EGCG is cleared from cells by 24 hours. BMDM treated with 1–5 μM EGCG showed no significant increase in LDH activity, whereas 10 μM and 25 μM EGCG resulted in ~5%, and ~ 20% cytotoxicity, respectively (Fig 4C). Pre-treatment with 5 μM EGCG decreased LPS-induced ROS generation by ~60–70%, measured by the DCF probe fluorescence (Fig 4D).

### EGCG alters dimerization and DNA binding activity of PU.1

**Fig 5:**
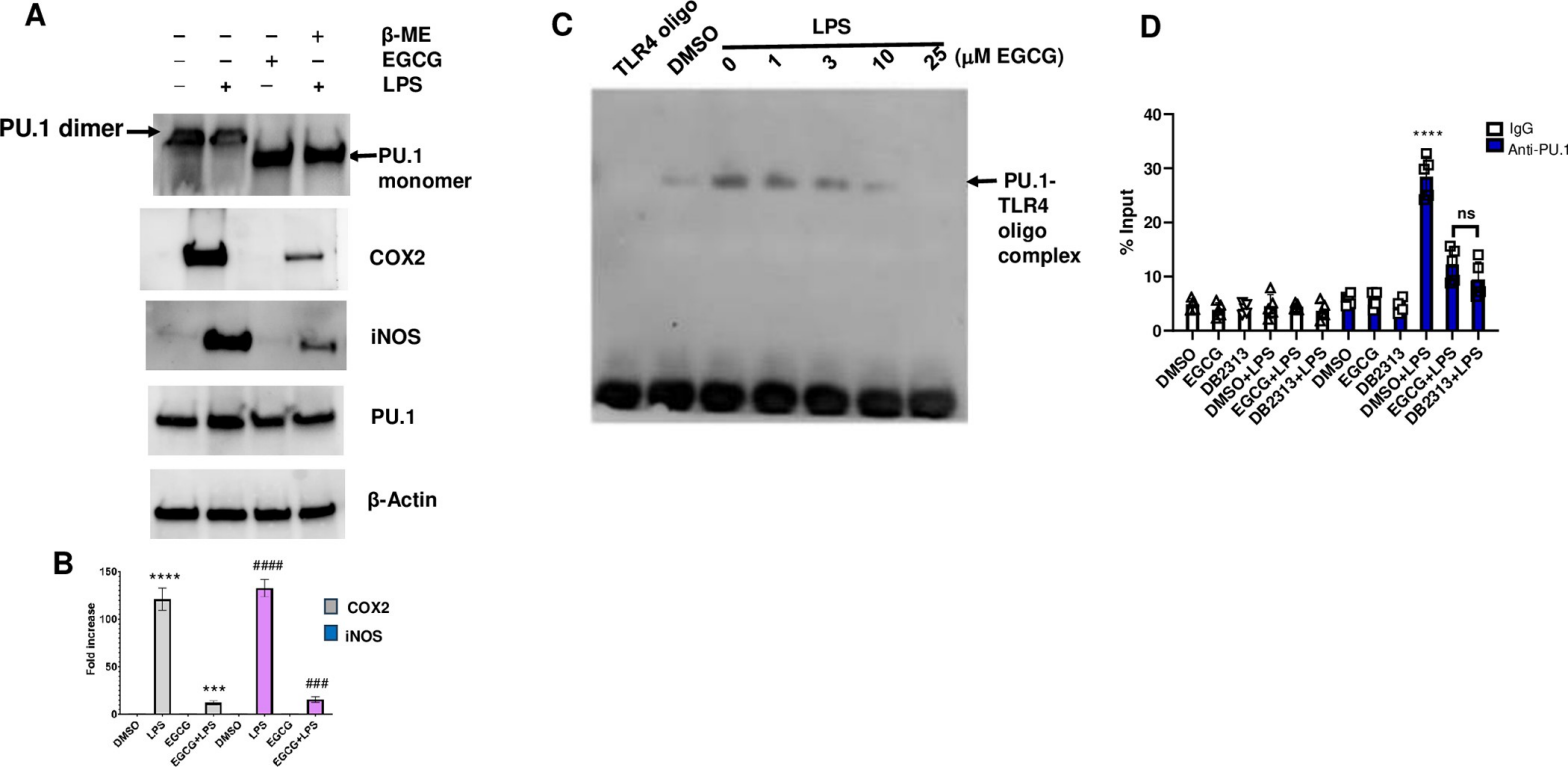
EGCG alters PU.1 dimerization and DNA binding activity. **A)** Total cell lysates from BMDM were incubated in presence of 25 μM EGCG and 1x β-Mercapto ethanol separately and immunoblotted with PU.1 antibody to determine the monomeric or dimeric state. Relative expression levels of COX2, iNOS and β-actin were determined in BMDM pretreated with EGCG, later stimulated with LPS, and **B)** relative densitometry. **C)** BMDM nuclear protein extracts were incubated with biotin labeled TLR4 promoter oligo in presence of increasing concentrations of EGCG. The reaction mix was electrophoresed on native polyacrylamide gel in 1X TBE, transferred to Biodyne A membrane and detected by Streptavidn-HRP chemiluminescence detection kit. **D)** BMDM were pretreated with 5 μM of EGCG or DB2313 for 1 h and stimulated with LPS or PBS for another 2 hours. Cells were cross-linked with 1% formaldehyde and PU.1 bound TLR4 promoter was immunoprecipitated, detected by qPCR after sequential washes, de-cross linking and purification steps. PU.1 recruitment on to TLR4 promoter was expressed as % input of chromatin. Graph shows means plus SD for triplicate samples and is representative of 2 independent experiments. Data are shown as mean ± SEM. N = 5 for each group, **D** **** p < 0.0001 Normal IgG immunoprecipitated chromatin vs Anti-PU.1 IgG immunoprecipitated chromatin.

Using mutational analysis, Esaki et al have demonstrated that PU.1 binds with DNA in different modes and it appears that PU.1 forms homodimers, indicated by interactions at W192, S184, W193, D197, B199 amino acids [44]. This study also demonstrates that, monomeric PU.1 has higher affinity to DNA binding than the dimeric form [44]. Glide docking shows that EGCG forms Hydrogen bonds with Lysine 196, Tryptophan 193, and Leucine 182 in the self-dimerization domain of PU.1 protein. To determine if binding of EGCG with PU.1 alters its oligomeric state, cytosolic protein from BMDMs was incubated with 25 μM EGCG (no heating, native condition) and β-mercapto ethanol (heated at 95°C for 2 minutes) separately, electrophoresed on native PAGE gels and immunoblotted with anti-PU.1 antibody. In the absence of β-mercaptoetanol or EGCG, PU.1 existed in dimeric form resulting in an immunoreactive band at ≥ 60 kDa region (Fig 5A). In contrast, 25 μM EGCG disrupted the PU.1:PU.1 dimeric conformation, resulting in a ~42 kDa immunoreactive PU.1 band (Fig 5A). Similarly, β-mercapto ethanol heating denatured the dimeric conformation resulting in monomeric PU.1, close to ~42 kDa. However, EGCG has no impact on total expression levels of cellular PU.1, but decreased the LPS-induced inflammatory proteins including, COX2 and iNOS (Fig 5A, densitometry shown in Fig 5B). Binding of PU.1 to 5′ Biotin-TLR4 promoter DNA was significantly decreased by treatment with EGCG in a dose dependent manner, and 10 μM EGCG decreased PU.1 binding to TLR4 promoter oligo, to a level comparable with that of controls (Fig 5C). Similarly, PU.1 binding to TLR4 promoter determined by ChIP showed ~5 fold increase in recruitment of PU.1 protein to TLR4 promoter, encompassing the PU.1 consensus sequence in -90 region (Fig 5D). As expected, pretreatment of BMDM with EGCG decreased LPS induced recruitment of PU.1 on to TLR4 promoter, indicating that EGCG is a potent small molecule inhibitor of PU.1-transcriptional activity (Fig 5C and 5D). % Input of immunoprecipitated TLR4 promoter DNA by control IgG/pre-immune serum or Anti-PU.1 precipitated control treatments (DMSO, EGCG, DB2313) is only ~ 5–6%, indicating specificity of PU.1 binding with DNA after LPS treatment (Fig 5D). As expected, the commercially available heterocyclic diamidine inhibitor of PU.1, DB2313, significantly inhibited PU.1 recruitment to TLR4 promoter (Fig 5D).

### EGCG inhibits PU.1 dependent macrophage gene expression

**Fig 6:**
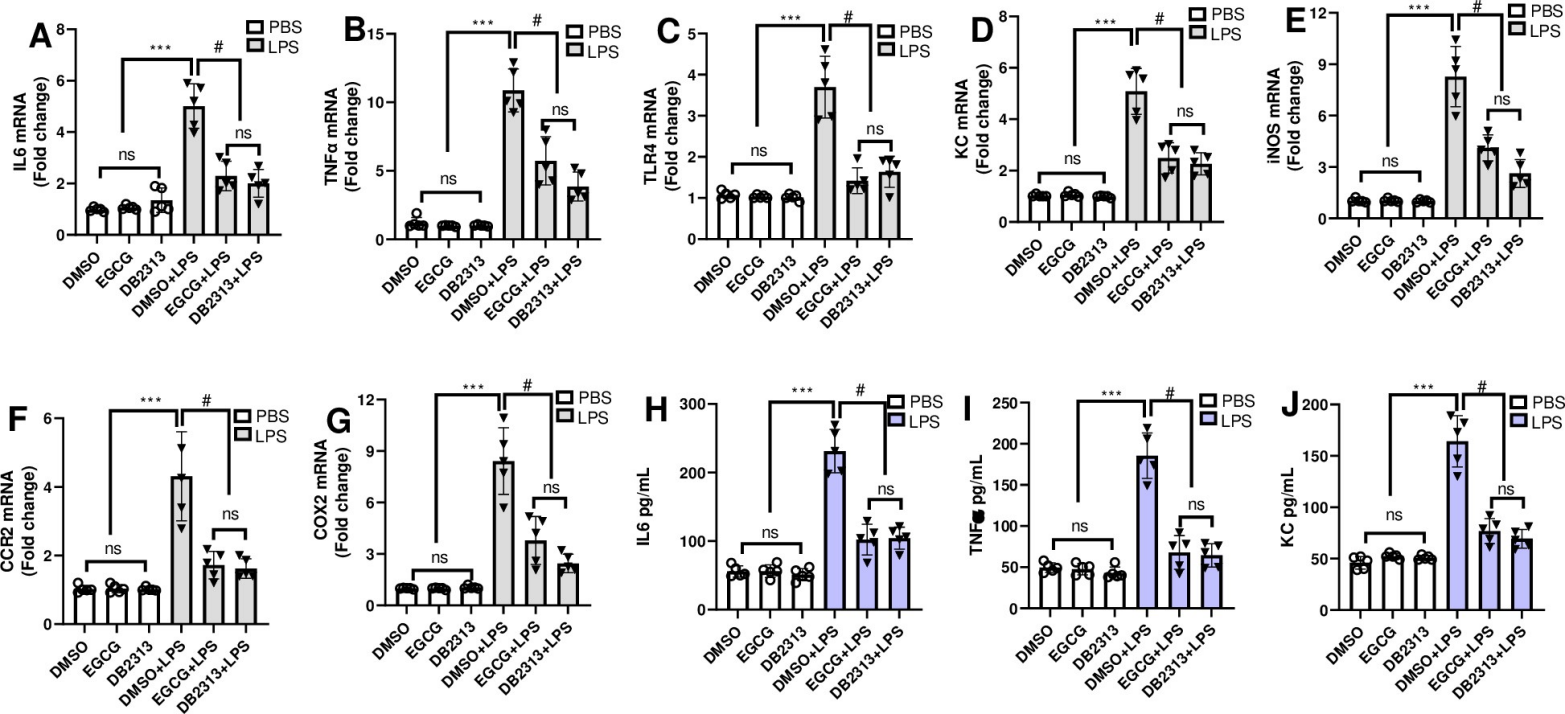
EGCG downregulates LPS-inducible PU.1 dependent macrophage gene expression. **A-G)** BMDM were pretreated with 5 μM of EGCG for 1 h and stimulated with LPS or PBS for another 4 hours. Expression of IL6, TNFα, TLR4, KC, iNOS, CCR2 and COX2 mRNA levels was determined using gene specific primers and SYBR green reaction mix, as described in methods. **H-J)** BMDM were pretreated with 5 μM of EGCG or DB2313 for 1 h and stimulated with LPS or PBS for another 16 hours and extracellular release of IL6, TNFα, and KC were determined by ELISA. Data are shown as mean ± SEM. N = 5 for each group, **A-G** ***p < 0.001 DMSO/EGCG/DB2313 vs DMSO+LPS; # p < 0.01 EGCG+LPS or DB2313+LPS or DMSO or EGCG or DB2313 vs DMSO+LPS. **H-J** ***p < 0.001 DMSO/EGCG/DB2313 vs DMSO+LPS; # p < 0.01 EGCG+LPS or DB2313+LPS vs DMSO+LPS.

**Fig 7:**
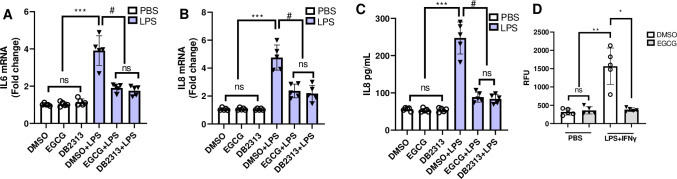
EGCG downregulates LPS-inducible PU.1 dependent genes in THP-1 cells. THP-1 Cells were pretreated with 5 μM of EGCG or DB2313 for 1 h and stimulated with LPS or PBS for another 4 hours. Expression of **A)** IL6 and **B)** IL8 mRNA levels was determined using gene specific primers and SYBR green reaction mix. THP-1 Cells were pretreated with 5 μM of EGCG or DB2313 for 1 h and stimulated with LPS or PBS for another 16 hours and extracellular release of **C**) IL8, and **D**) ROS generation were determined by ELISA and DCFH-DA dye. **A-D** Data are shown as mean ± SEM. N = 5 for each group, **A-C** *** p < 0.001 DMSO/EGCG/DB2313 vs DMSO+LPS; # p < 0.01 EGCG+LPS or DB2313+LPS or DMSO or EGCG or DB2313 vs DMSO+LPS. **D** ** p < 0.01 DMSO/EGCG vs DMSO+LPS; * p < 0.05 EGCG+LPS vs DMSO+LPS.

To determine whether EGCG alters transcriptional target genes of PU.1, we have measured key inflammatory genes in mouse BMDM and THP-1 monocytes by quantitative PCR and ELISA. LPS increased expression levels of IL6, TNFα, TLR4, KC, CCR2, iNOS and COX2 mRNA in mouse BMDM. The fold increase varied from ~4–10 for different genes. Pretreatment with 5 μM EGCG for 1 hr significantly decreased LPS induced expression of IL6, TNFα, TLR4, KC, iNOS, CCR2, and COX2 mRNA levels (Fig 6A–6G). Similar to the mRNA levels, the extracellular release of IL6, TNFα, KC by BMDMs followed same pattern as that of mRNA levels indicating the inhibitory effect of EGCG on inflammatory gene expression (Fig 6H–6J). Included as a positive control, DB2313 down regulated all the PU.1 target genes similar to EGCG (Fig 6A–6J). As the mouse and human PU.1 proteins show high sequence homology in both the DNA binding and dimerization domains, we expected EGCG to show similar activity in human macrophages. To determine this, THP-1 differentiated macrophages pretreated with 5 μM EGCG and stimulated with LPS and were analyzed. LPS has increased expression levels of IL6, IL8 mRNA, extracellular release of IL8, which were attenuated by EGCG and DB2313 pre-treatment (Fig 7A–7C). As observed in mouse BMDM, EGCG decreased LPS-induced ROS generation in THP-1 cells (Fig 7D).

## Discussion

Our previous studies show that functional inactivation of PU.1 results in marked attenuation of lung and systemic inflammation in mouse models of sepsis [23]. PU.1-deficient mice showed reduced lung inflammation parameters in a high-dose endotoxin-induced sepsis model, indicating beneficial effect of PU.1 inactivation in severe sepsis and multiple organ failure cases. In the current study, we have identified EGCG as a ligand that specifically binds with PU.1 protein and inhibits its transcriptional activity. As the green tea polyphenols are routinely used as dietary supplements, EGCG associated systemic toxicity is not anticipated [45].

*In silico* molecular docking analysis using Glide indicates that EGCG interacts with both DNA binding and PU.1 homo-dimerization domains within the protein, which is in agreement with published results on the oligomeric state of PU.1 [44]. However, the *insilico* approach has certain limitations, where docking analysis may identify multiple proteins that bind with the same ligand. For example, EGCG was shown to bind with proteins such as protease activated receptor 2, matrix metalloproteinase 2, and receptor tyrosine kinases [40,46,47]. The most probable reasons for this docking promiscuity include structural similarity between target proteins, ligand structural flexibility, or computational limitations. To overcome these limitations, we have determined the cellular activity of EGCG by in vitro experimental analysis of primary macrophages and THP-1 cells.

*In vitro* experiments confirmed that EGCG-TRITC is taken up by mouse BMDM in a time dependent manner. The calculated dissociation constant of PU.1-EGCG-TRITC binding by FA assay was 2.8 μM indicating a strong protein-ligand interaction. Based on experimental analysis, we found that EGCG has an inhibitory effect on the transcriptional and DNA binding activity of PU.1, particularly in the TLR4 promoter region. In mouse BMDMs, TLR4 dependent inflammatory genes were significantly downregulated by both DB2313 and EGCG. TLR4, KC, IL6, TNFα, iNOS and COX2 were reported as transcriptional targets of PU.1 by our team and few other research groups previously [13,16,23]. It is important to note that EGCG has no impact on total PU.1 expression in macrophages but only decreased expression of inflammatory genes induced by LPS.

Small molecule inhibitors of PU.1 protein developed by Munde et al., have been shown to interact with PU.1 ETS/DNA binding [25,26]. Subsequent studies have confirmed the heterocyclic di-amidines and heterocyclic di-cations as small molecule inhibitors that bind with AT rich DNA minor grooves, thereby inhibit PU.1-DNA binding [27,28]. The small molecule inhibitors disrupt the interaction of PU.1 with target gene promoters and downregulate expression of PU.1 transcriptional targets. In murine and human AML (xeno) transplantation models, treatment with heterocyclic di-amides decreased tumor burden and resulted in increased survival [48]. In addition, these compounds have demonstrated therapeutic potential in different animal fibrosis models [49–51]. Similarly, our studies demonstrate that EGCG inhibits inflammatory gene expression in mouse macrophages and human monocytes. In summary, we demonstrate by using both docking studies and experimental verification, that EGCG binds to PU.1 protein and downregulates several inflammatory genes in macrophages. Based on current data, EGCG has potential to block inflammatory gene expression in macrophages and may hold therapeutic potential for treating inflammatory lung diseases involving these cells.

## Conclusion

Our study has identified a novel mechanism of EGCG-PU.1 interaction in mouse and human macrophages. EGCG binds to PU.1 protein, altering its transcriptional activity in macrophages implicating the therapeutic potential of EGCG for mitigating inflammatory lung diseases.

## Supporting information

S1 File Contains 4. Representative data sets.xls (for Figs 3–7) 5. uncropped western blots.pptx (for Fig 5A).
(ZIP)

S1 Fig (TIF)
