# Supplementary figures and images for: Epigallocatechin gallate regulates the myeloid-specific transcription factor PU.1 in macrophages
Source: PLoS One. 2024 Apr 25;19(4):e0301904. doi: 10.1371/journal.pone.0301904 (PMC11045095; doi:10.1371/journal.pone.0301904)

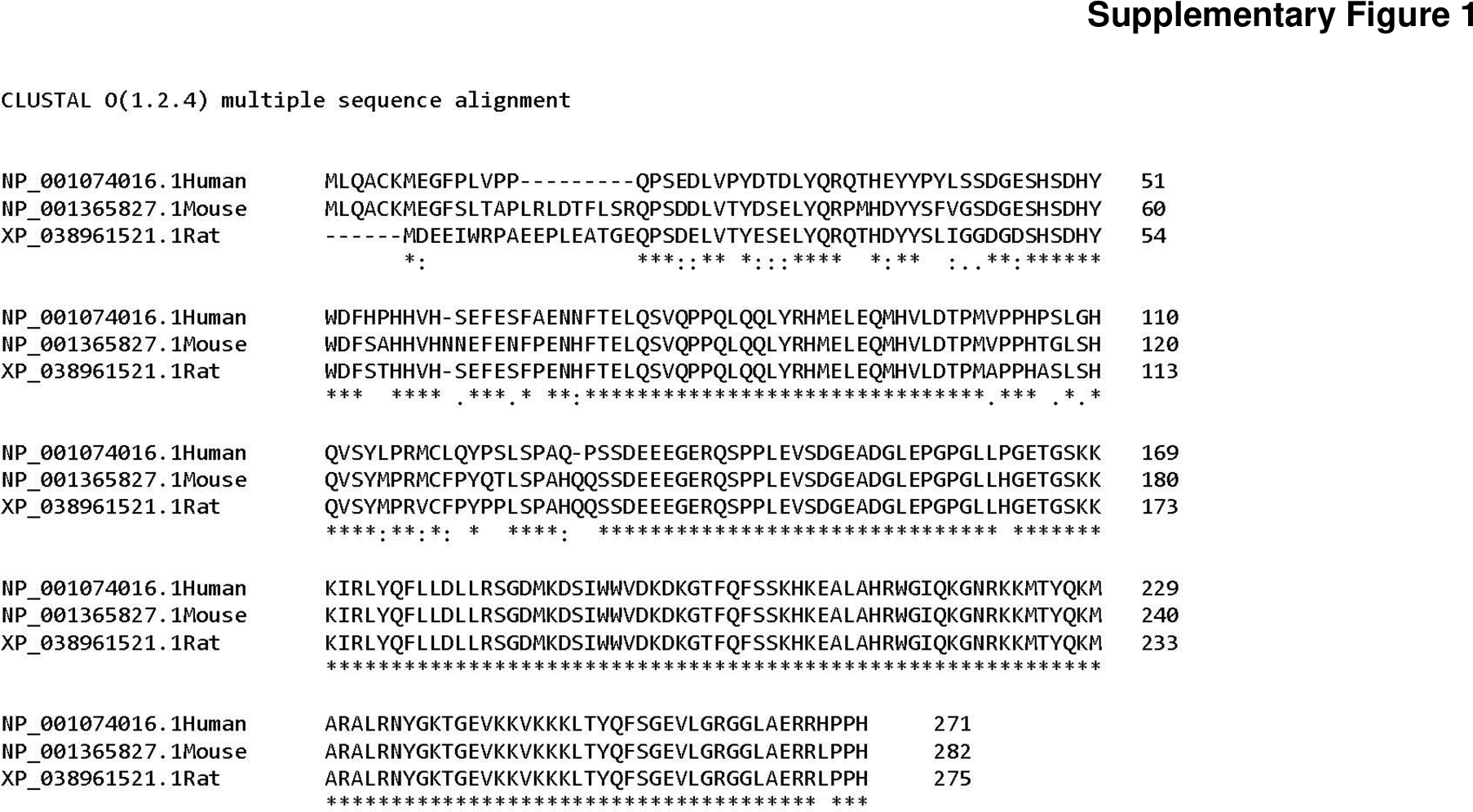

Supplement: S1 Fig — (TIF) [file pone.0301904.s002.tif]
